# Induction of microRNA resistance and secretion in differentiating human endometrial stromal cells

**DOI:** 10.1093/jmcb/mjs058

**Published:** 2012-10-25

**Authors:** Kunal M. Shah, Jason Webber, Raffaella Carzaniga, Deborah M. Taylor, Luca Fusi, Aled Clayton, Jan J. Brosens, Geraldine Hartshorne, Mark Christian

**Affiliations:** 1 Division of Surgery and Cancer, Institute of Reproductive and Developmental Biology, Imperial College London, Hammersmith Campus, London W12 0NN, UK; 2 Institute of Cancer and Genetics, School of Medicine, Cardiff University, Velindre Cancer Centre, Whitchurch, Cardiff CF14 2TL, UK; 3 Electron Microscopy Centre, Biochemistry Building, Imperial College London, South Kensington Campus, London SW7 2AZ, UK; 4 Division of Reproductive Health, Warwick Medical School, University of Warwick, Coventry CV4 7AL, UK

Dear Editor,

A key event in the preparation for pregnancy is the differentiation of human endometrial stromal cells (hESCs) into epithelioid decidual cells. This process, termed decidualization, is initiated in the stromal cells of the superficial endometrial layer ∼9 days after ovulation. Upon decidualization, hESCs acquire the ability to resist oxidative stress, to modulate immune responses to fetal alloantigens, and to control trophoblast invasion. Differentiating primary hESCs recapitulate many changes in gene expression observed upon decidualization *in vivo* with progesterone and cAMP signaling pathways cooperating in hESCs at several levels [see review (Gellersen and Brosens, 2003)].

MicroRNAs (miRNAs) are a diverse class of non-coding small RNAs, which post-transcriptionally regulate gene expression by interacting with sites in the 3′UTRs of mRNAs. miRNA synthesis begins with the transcription of miRNA genes to produce primary miRNAs (pri-miRNAs). In the nucleus, the pri-miRNAs are cleaved by the Drosha/DGCR8 complex to produce ∼70 nt pre-miRNAs. Exportin-5 transfers pre-miRNAs to the cytoplasm where Dicer removes the loop to give duplex mature miRNA of ∼22 nt. Argonaute proteins facilitate the miRNA–mRNA interactions and mediate silencing. In addition to their diverse roles in the parental cell, miRNAs can act as paracrine or endocrine signals and are present in the plasma as well as in the conditioned medium of cultured cells (Kosaka and Ochiya, 2011).

Endometrial miRNA expression is cycle dependent and regulated by ovarian hormones (Pan et al., 2007). With miRNA microarray analysis we identified 16 significantly (*P* < 0.05) differentially expressed miRNAs in primary hESCs that had been vehicle treated or decidualized with 8-Br-cAMP and medroxyprogesterone acetate (C + M) for 8 days (Supplementary Table S1). Real-time qPCR was used to monitor the expression of four miRNAs (miR-29b, miR-29c, miR-100, and miR-143) in independent primary hESC cultures treated either with vehicle or with C + M for 2, 4, or 8 days. These miRNAs were selected for study as they showed the most significant changes in expression and were likely to target genes important for decidual function including DNA methyltransferase 3b (DNMT3B), Tribbles 2 (TRIB2) and prokineticin 1 (PROK1). Figure 1A shows that the expression of miR-29b, miR-29c, and miR-100 increases as expected by 8 days of C + M treatment. In contrast, the levels of mature miR-143 gradually declined, which is also in keeping with the array data. The changes in the pri-forms of the four miRNAs during decidualization largely mirrored the changes observed in mature miRNA levels (Supplementary Figure S1), indicating that transcriptional regulation of miRNA genes occurs during decidualization.

**Figure 1:**
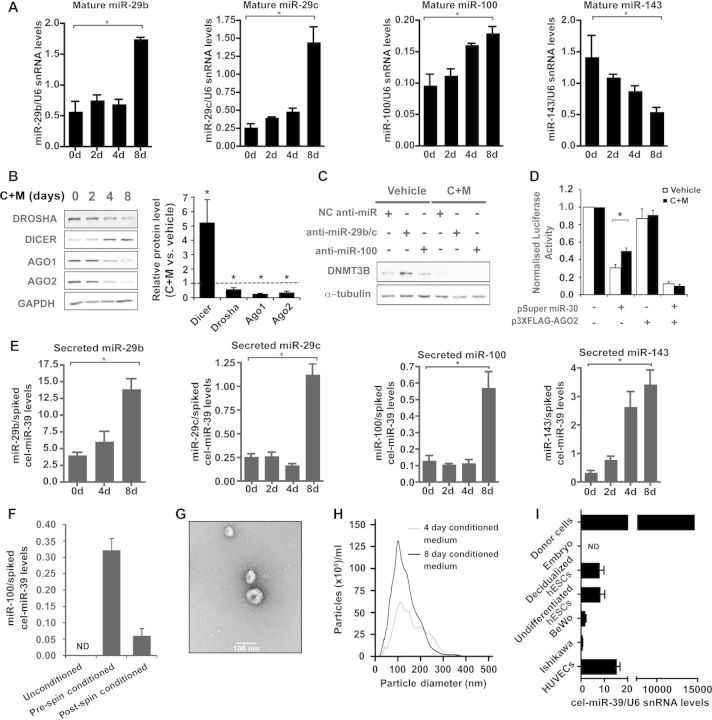
Decidualization leads to differential miRNA expression, miRNA pathway regulation and miRNA secretion. (**A**) Primary hESCs were vehicle treated (day 0) or treated with C + M for 2, 4, or 8 days. qPCR was performed for mature miR-29b, miR-29c, miR-100, and miR-143. miRNA levels were normalized U6 snRNA levels. Data show the mean of *n* = 3 and error bars denote the SEM. *P < 0.05. (**B**) Western blots of miRNA pathway components in hESCs treated with vehicle or C + M for 2, 4, and 8 days. GAPDH acted as a loading control. The graph shows densitometric analysis of three independent blots comparing vehicle treated and day 8 C + M treated samples. Vehicle-treated levels were normalized to 1. *P < 0.05. (**C**) hESCs were transfected with negative control (NC) anti-miR or anti-miR-29b/c or anti-miR-100 and treated with vehicle or C + M for 2 days before harvesting and western blotting for DNMT3B. Alpha-tubulin acted as a loading control. (**D**) hESCs in 24-well plates were transfected with pCMV Luc miR-30-(P) (300 ng/well), pcH110, 20 ng/well of miR-30, and 50 ng/well p3XFLAG-AGO2. hESCs were treated with vehicle or C + M for 3 days before harvesting. Luciferase activity levels were normalized to beta-galactosidase levels. Data represent fold change relative to the reporter-only sample. Each bar denotes the mean of four replicates and the error bars represent the SEM. *P < 0.05. (**E**) Cultured hESCs were treated with vehicle (day 0) or C + M for 2, 4, and 8 days. For the final 2 days before harvesting, cells were incubated in serum-free medium. qPCR was used to profile expression of miR-29b, miR-29c, miR-100, and miR-143 in conditioned media. miRNA expression levels were normalized to levels of spiked cel-miR-39. Data shows the mean of three replicates and the error bars represent the SEM. *P < 0.05. (**F**) Serum-free conditioned medium from decidualized hESCs was serially centrifuged and the supernatants were used immediately (pre-spin) or ultracentrifuged (postspin). qPCR was performed for miR-100. Unconditioned media was also tested in parallel. Bars show miR-100 levels normalized to spiked cel-miR-39 levels for triplicate samples. Error bars denote SEM. ND indicates not detected. (**G**) Transmission electron micrograph of vesicles obtained by ultracentrifugation of serum-free conditioned medium from decidualized hESCs. (**H**) Nanosight size profiling of particles in decidual cell conditioned media. (**I**) Uptake of cel-miR-39 by embryos and cells from conditioned medium of decidualized hESCs transfected with pSuperior cel-miR-39. Cel-miR-39 levels were normalized to endogenous U6 snRNA levels. Bars represent measurements for triplicates and error bars denote SEM (see Supplementary Method).

Next, we examined whether key components of the miRNA synthesis pathway are altered upon decidualization of hESCs. Parallel primary cultures treated with C + M for various time points were harvested for total protein and RNA. The levels of Drosha, Dicer, Ago1 and Ago2 were assessed at the protein and transcript levels (Figure 1B and Supplementary Figure S2). Drosha levels modestly decline in response to C + M treatment. Conversely, Dicer is up-regulated and again this response seemed more pronounced at the protein level. Strikingly, C + M treatment profoundly inhibited the abundance of Ago1 and Ago2, especially at the later time points. Densitometric analysis comparing three independent samples from day 0 and day 8 confirmed the changes observed (Figure 1B). siRNA-mediated knockdown of Dicer did not block the induction of the classical decidual marker genes prolactin (PRL) and insulin-like growth factor binding protein 1 (IGFBP1) during decidualization (Supplementary Figure S3). The robust induction of these genes indicated that Dicer-depleted hESCs are still able to differentiate upon treatment with C + M. This result is in keeping with the data from the conditional Dicer KO mouse where female mice lacking Dicer in the reproductive tract were infertile due to defective oviducts but exhibited histologically normal decidual responses (Nagaraja et al., 2008).

Although Dicer knockdown did not block hESC differentiation, we speculated that regulated miRNAs target and modulate the expression of specific decidual genes. An experimentally validated target of the miR-29 family is DNMT3B (Fabbri et al., 2007). To test if DNMT3B expression is inhibited upon decidualization as a consequence of miR-29b/c induction, primary cultures were first transfected with anti-miR-29b/c and then treated with vehicle or C + M. As shown in Figure 1C, DNMT3B expression is indeed down-regulated upon decidualization at the protein level. Furthermore, the transfection of anti-miR-29b/c enhanced DNMT3B protein levels in undifferentiated cells, although this response was entirely lost upon treatment with C + M. In decidualizing hESCs, the effect of anti-miR-29b/c transfection on DNMT3B expression was indistinguishable from transfection negative control anti-miR or non-targeting anti-miR-100. Similar findings were also observed with respect to DNMT3B mRNA levels, for TRIB2, a predicted target of both miR-29b/c and miR-100, and for PROK1, a predicted target of miR-100 (Supplementary Figure S4).

We employed a reporter assay to monitor the generic miRNA activity in both undifferentiated and decidualized hESCs. Primary cultures were co-transfected with pCMV Luc miR-30-(P), a reporter construct containing eight miR-30-binding sites in the 3′UTR (Zeng et al., 2003), and pSuper miR-30. The co-expression of pSuper miR-30 inhibited luciferase activity in both vehicle and C + M treated cells (Figure 1D). However, miR-30-dependent repression was significantly higher in undifferentiated compared with decidualizing hESCs. Furthermore, the co-expression of Ago2 in decidualizing cells restored the level of miR-30-dependent repression to that observed in undifferentiated cells. Together, the data show that down-regulation of Ago2 induces resistance to endogenous miRNA-dependent gene repression in decidualizing cells. Although previous studies found that Ago-deficient mouse embryonic stem cells were more apoptotic (Su et al., 2009), this is unlikely to be the case for hESCs as C + M treatment renders hESCs more resistant to apoptosis (Kajihara et al., 2006). Therefore, the resistance to miRNA function observed in decidualizing hESCs is unlikely to be a secondary effect of increased apoptosis. During decidualization, the cAMP dependent up-regulation of FOXO1 is important for cell cycle regulation and conferring resistance to oxidative stress, as well as the induction of differentiation markers such as IGFBP1 (Lam et al., 2012). It has also been documented in endometrial cancer that the miR-27 family can reduce FOXO1 expression (Myatt et al., 2010). We observed a down-regulation of miR-27b upon decidualization (Supplementary Table S1) and this could contribute to the increase in FOXO1 levels that are crucial for several decidual cell phenotypes, including resistance oxidative stress-induced apoptosis.

Decidualizing hESCs acquire a secretory phenotype (Salamonsen et al., 2007). As differentiation also bestows resistance to miRNA-mediated gene silencing, we speculated that decidual miRNAs may be intended for export. To test this hypothesis, primary hESCs were decidualized with C + M for various time points. The cultures were maintained in serum-free medium for 48 h prior to harvesting. RNA was then extracted from the conditioned medium and miR-29b, miR-29c, miR-100, and miR-143 amplified by qPCR (Figure 1E). The abundance of the four miRNA species in the spent media was uniformly higher by 8 days of C + M treatment. Notably, the rise in miR-143 secretion appears to be an early event in the decidual process. This is particularly striking as the cellular levels of both the pri- and mature forms of this miRNA decline in response to C + M treatment (Figure 1A and Supplementary Figure S1). The data suggest that miR-143 may be stored in undifferentiated hESCs and readily released upon decidualization.

For secretion, miRNA must be packaged in membrane-bound vesicles, known variously as microvesicles, exosomes, and large membranous vesicles. Ultracentrifugation of serum-free conditioned medium depleted miR-100 levels in the supernatant but miR-100 was abundant in the resultant pellet (Figure 1F and Supplementary Figure S5A). Western blotting showed that the ultracentrifugation pellet was enriched in exosome markers TSG101 and HSP90 (Supplementary Figure S5B). The enrichment of small RNA in the ultracentrifugation pellets was confirmed using an Agilent Bioanalyzer (Supplementary Figure S5C).

Ultracentrifugation at 100000 *g* is routinely used to pellet exosomes (Thery et al., 2006), and transmission electron microscopy revealed that the pellets from decidual cell conditioned medium contained cup-shaped vesicles that measured <100 nm in diameter, indicative of exosomes (Figure 1G). Conditioned medium that had been incubated with decidualized hESCs for 4 or 8 days was analyzed using the Nanosight nanoparticle tracking system (Figure 1H). The size profiles of particles in decidual cell conditioned media were typical of samples rich in exosomes (Vallhov et al., 2011), with a large proportion of particles having a diameter of 100 nm or less. Larger particles are likely to be aggregates of exosomes.

We sought to test if human embryos and other cell types present at the feto-maternal interface could take up secreted decidual miRNAs. We transfected hESCs with pSuperior cel-miR-39, which expresses a miRNA without mammalian orthologs, and treated the cells with C + M for 6 days. The conditioned medium was collected and added to a number of relevant cell lines, including Ishikawa cells (representing endometrial epithelial cells), BeWo cells (a trophoblast cell line), and human umbilical vein endothelial cells (HUVECs). Human embryos and hESCs, decidualized or not, were also tested as recipients. Where embryos were used as recipients, the conditioned medium was ultracentrifuged and the pellet was resuspended in embryo culture medium. Following exposure to conditioned medium, cel-miR-39 was detected in HUVECs, BeWo cells, and hESCs and, to a lesser extent, in Ishikawa cells (Figure 1I). Although we were able to amplify endogenous U6 snRNA in human embryos, cel-miR-39 was not detectable. However, this finding must be considered preliminary in view of the presence of the zona pellucida in some of our cultured embryos, which could have prevented uptake, and those embryos with breached zonas failed to develop into blastocysts. HUVECs contained the highest levels of cel-miR-39, which were ∼1000-fold less abundant than in the donor hESCs that had been transfected with pSuperior cel-miR-39. We did not observe a significant difference in cel-miR-39 uptake between undifferentiated and decidualized hESCs. Thus, it appears likely that various cells that make up the feto-maternal interface take up miRNAs secreted by decidualized hESCs.

In summary, our results show that miRNA signaling is profoundly altered during decidualization of hESCs in preparation for pregnancy. The physiological relevance of diminished miRNA silencing in decidualized hESCs may be a defence mechanism to protect from the silencing effects of trophoblast-derived miRNAs. A recent study found that placenta-specific miRNAs were abundant in the circulation of pregnant women and trophoblast cells secreted miRNAs in exosomes (Luo et al., 2009). Our data also provide the first evidence that miRNAs can be added to the growing number of factors known to be secreted by decidual cells and their uptake by other cells of the endometrium may have physiological consequences that remain to be explored.

*[Supplementary material is available at Journal of Molecular Cell Biology online*. *We thank Nick Dibb (Imperial College London) for advice on the manuscript. This work was supported by BBSRC grant (BB/D52657X/1), the Genesis Research Trust, and the Biomedical Research Unit in Reproductive Health, a joint initiative between the University Hospitals Coventry and Warwickshire NHS Trust and Warwick Medical School.]*

## Supplementary Material

Supplementary Data
