# Supplementary material for: Induction of microRNA resistance and secretion in differentiating human endometrial stromal cells
Source: J Mol Cell Biol. 2012 Oct 25;5(1):67–70. doi: 10.1093/jmcb/mjs058 (PMC3755475; doi:10.1093/jmcb/mjs058)
Supplement: Supplementary Data [file supp_5_1_67__index.html]

Induction of microRNA resistance and secretion in differentiating human endometrial stromal cells — Induction of microRNA resistance and secretion in differentiating human endometrial stromal cells — Induction of microRNA resistance and secretion in differentiating human endometrial stromal cells — Supplementary Data 

# Induction of microRNA resistance and secretion in differentiating human endometrial stromal cells

## Supplementary Data

Supplementary Data

**Files in this Data Supplement:**

- Supplementary Data - Pdf file
