# Supplementary material for: Induction of microRNA resistance and secretion in differentiating human endometrial stromal cells
Source: J Mol Cell Biol. 2012 Oct 25;5(1):67–70. doi: 10.1093/jmcb/mjs058 (PMC3755475; doi:10.1093/jmcb/mjs058)
Supplement: Supplementary Data [file supp_mjs058_mjs058supp.pdf]

Supplementary Materials & Methods

Endometrial stromal cell culture

Endometrial biopsies were obtained by curettage from consenting women aged 18-40 in accordance with local ethics committee requirements. Endometrial stromal cells were isolated and cultured as described previously (1). All experiments were performed within the first three cell passages. To induce decidualization *in vitro,* hESCs were treated with 0.5 mM 8-Bromo-cAMP (Sigma, St. Louis, MO, USA) and 1 μM medroxyprogesterone acetate (MPA) (Sigma) in DMEM/F12 medium supplemented with 2% dextran-coated charcoal (DCC) treated fetal bovine serum (FBS), L-glutamine and antibiotic-antimycotic (Invitrogen, Paisley, UK). Where hESCs were treated in serum-free medium, all supplements, excluding DCC-FBS, were added to the medium. Vehicle treatments consisted of absolute ethanol at an equivalent concentration. Culture medium was normally replenished every two days.

*Cell line cultu*re

BeWo and Ishikawa cells were maintained in DMEM/F12 medium supplemented with 10% fetal bovine serum (FBS), L-glutamine and antibiotic-antimycotic (Invitrogen) HUVECs were maintained in Ham's F12 medium (Invitrogen) supplemented with 10% FBS, sodium bicarbonate, L-glutamine, antibiotic-antimycotic, heparin and endothelial cell growth supplement (Sigma).

Human oocytes and embryos

Women underwent ovarian stimulation and oocytes were collected by transvaginal ultrasound-guided aspiration and inseminated with prepared sperm (day 0). Oocytes were examined 19 - 20 hours after insemination, and classified as normally fertilised if two pronuclei were present. Fertilised embryos were cultured in MediCult media (Origio Ltd, Reigate, UK) to day 5 of development. Following embryo transfer, surplus embryos were donated to research by couples who gave informed consent. This work was carried out under a Human Fertilisation and Embryology Authority research licence, with approval from the Local Research Ethics Committee.

Embryo culture

Eleven human embryos were used in this study, three at the blastocyst stage and 8 that had arrested development by day 5. Three of the embryos had holes made in the zona

pellucida as part of a separate experiment. Embryos were cultured individually in 50 μl drops of MediCult BlastAssist medium (Origio) containing ultracentrifugation extracts from decidual cell conditioned medium. Following overnight culture (16 hours) embryos were washed through several drops of PBS prior to the complete removal of the zona pellucida with acidified Tyrode's solution. Denuded arrested embryos and blastocysts were again washed in PBS, then individual embryos were lysed in 10μl of Lysis solution of the Single Cell Lysis Kit, (Ambion, Life Technologies, Carlsbad, CA, USA) and stored at -20°C.

miRNA Microarray

To isolate small RNAs, 10 μg of total RNA from each sample was processed using the PureLink miRNA Isolation kit (Invitrogen) according to the manufacturer's instructions. Using the NCode miRNA Rapid labelling system (Invitrogen), a poly(A) tail was added and an Alexa Fluor 3-labelled DNA polymer was ligated to the RNA. Labelled RNAs were then hybridized to NCode multispecies miRNA microarrays V2 (Invitrogen) and scanned using an Agilent microarray scanner (Agilent, Santa Clara CA, USA). Microarray images were analysed using Agilent's Feature Extraction Software and statistical analysis was performed with GeneSpring GX software (Agilent).

Transient Transfection and Plasmid Constructs

Primary hESCs were transfected by the calcium phosphate co-precipitation method using the ProFection Mammalian Transfection kit (Promega, Madison, WI, USA). For anti-miR experiments, miScript miRNA Inhibitors and Negative Control were purchased from Qiagen (Crawley, UK). Plasmid constructs for the reporter assay, pCMV Luc miR-30 (P) and pSuper-miR-30, were kindly provided by Bryan Cullen (Duke University, Durham, NC, USA). p3XFLAG-AGO2 was kindly provided by Nick Dibb. An oligonucleotide containing the sequence for cel-miR-39 was cloned into the HindIII and BglII sites of pSuperior.puro vector (Oligoengine, Seattle, WA, USA).

Reporter Assays

For reporter assays, hESCs were harvested in 1x Reporter Lysis Buffer (Promega, Madison, WI USA) as recommended by the manufacturer. Luciferase Assay Reagent

(Promega) was added to lysates and luciferase activity was measured using a Wallac Victor 1420 plate reader (Perkin-Elmer, Waltham, MA, USA). To normalize luciferase activity readings, a construct encoding beta-galactosidase (pcH110) was also transfected and beta-galactosidase activity was assayed in lysates using the Galacto-Light Plus kit (Applied Biosystems, Foster City, CA, USA).

RNA extraction from cells and media

Total RNA from cells was extracted using TRI Reagent (Sigma) according to the manufacturer's instructions. For RNA extraction from culture medium, conditioned media were collected and centrifuged at 300 x *g* for 10 min at room temperature, and the supernatant was then centrifuged at 12,000 x *g* for 30 min at 4 ^{o}C to remove all cell debris. RNA was extracted from the supernatants using TRI reagent LS (Sigma) and GlycoBlue was added as a co-precipitant (Ambion). Prior to RNA extraction, medium was spiked with 2 μl of a 1 nM solution of synthetic cel-miR-39 (Qiagen).

mRNA Reverse Transcription and Quantitative PCR

Prior to reverse transcription, RNA was treated with amplification-grade DNase I (Sigma). 20 μl reverse transcription reactions were set up using dNTPs, 5x First strand buffer, random hexamers, 0.1M DTT and MMLV reverse transcription (all reagents from Invitrogen) according to the manufacturer's instructions. Real time quantitative (q)PCR was performed with SYBR Green Jumpstart 2x Taq Readymix (Sigma) and specific primers using ABI StepOne Plus qPCR cycler (Applied Biosystems, Cheshire, UK). QPCRs were performed in duplicate for each sample. The target transcript levels were quantified by the relative standard curve method and normalized to L19 levels.

miRNA Reverse Transcription and Quantitative PCR

MiRNAs were reverse transcribed using the stem-loop RT primer system developed by Chen et al (2). Briefly, DNase-treated total RNA was mixed with a pool of miRNA stem-loop RT primers for several different miRNAs (each at a final concentration of 5 nM), dNTPs, 5x First Strand buffer, 0.1M DTT and MMLV reverse transcriptase. Reverse transcription reactions were performed with the pulsed cycle used by Tang et al. (3).

miRNA qPCR was performed with a universal reverse primer, a miRNA specific forward primer and SYBR Green Jumpstart 2x Taq Readymix (Sigma). For normalisation of cellular miRNA levels, U6 snRNA levels were also assayed for each sample. Relative standard curve analysis was used to quantify the abundance of PCR product.

For microRNA transfer experiments, cel-miR-39 was reverse transcribed as above but with 50 nM stem loop RT primer and U6 reverse primer. cDNA was pre-amplified prior to qPCR using a specific cel-miR-39 forward primer, universal reverse primer and Platinum Taq Supermix (Invitrogen). Pre-amplification PCR cycle conditions were 94 ^{o}C for 2 min followed by 16 cycles of 94 ^{o}C for 15 s, 55 ^{o}C for 20 s and 72 ^{o}C for 30 s. Pre-amplified cDNA was assayed for cel-miR-39 by qPCR using the specific forward primer, universal reverse primer and a specific Taqman probe and Sigma Jumpstart Taq 2x Readymix. Cel-miR-39 levels were normalised to endogenous U6 snRNA levels.

Western Blotting

Total protein was harvested on ice from hESCs in RIPA buffer (150 mM NaCl, 50 mM Tris pH 8.0, 1% NP-40, 5 mM EDTA, 0.5% deoxycholate, 0.1% SDS, 1mM PMSF, 1X complete Protease Inhibitor cocktail (Roche, Penzberg, Germany)) and cell lysates were centrifuged at 12,000 x *g* for 5 min at 4 ^{o}C. Supernatants were then collected, snap frozen and stored at -80 ^{o}C. Protein concentrations were determined with a BCA assay kit (Pierce, Rockford, IL, USA) according to the manufacturer's instructions. Proteins were resolved on polyacrylamide gels and electroblotted onto polyvinylidenedifluoride membranes (Millipore, Billerica, MA USA). Membranes were blocked for at least 1h in 5% non-fat milk before probing with primary antibody. Primary antibodies used in this study were rabbit anti-DICER, rabbit anti-DROSHA (used at 1:2000; Cell Signalling Technology, Danvers, MA, USA), rabbit anti-DNMT3B (used at 1:1000; Cell Signalling Technology), rat anti-AGO1, rat anti-AGO2 (kind gifts of Nick Dibb; used at 1:50), rat-anti α-tubulin (used at 1:2000; Abcam, Cambridge, UK), mouse anti-β-actin (used at 1:100,000; Abcam), mouse anti-GAPDH (used at 1:10,000; Millipore). Secondary antibodies used were HRP-conjugated anti-rat (Invitrogen), anti-mouse and anti-rabbit (used at 1:4000; Dako UK Ltd, Ely, UK). Membranes were probed with ECLPlus (GE Healthcare, Chalfont St.

Giles, UK) or ECL (Pierce) and imaged using an ImageQuant imager (GE Healthcare).

Ultracentrifugation of cell culture supernatants

Serum-free conditioned medium was collected from hESCs and serially centrifuged; first at 300 x *g* for 10 min at room temperature and then the supernatant was transferred to new tubes and centrifuged at 12,000 x *g* for 30 min at 4^oC to remove all cell debris. The supernatant was then ultracentrifuged at 100,000x*g* for 70 min at 4^oC in a SW28 swing-bucket rotor (Beckman Coulter, High Wycombe, UK). Pellets were resupended in 0.22 µm filtered PBS and ultracentrifugation repeated. Supernatants were removed and pellets were lysed in TRI Reagent for RNA extraction.

Electron microscopy of isolated vesicles

Ultracentrifugation pellets from serum-free conditioned medium were resuspended in PBS and ultracentrifugation was repeated. The resulting pellet was then resuspended in Tris-buffered saline and a small sample was loaded onto continuous carbon 300 mesh copper grids. After staining with a 2% aqueous solution of uranyl acetate, images were acquired using a Tecnai transmission electron microscope (FEI, Eindhoven, Netherlands) at an operating voltage of 120kV. Images were recorded using an Eagle CCD camera (FEI).

Nano-particle quantitation and sizing.

For nanosight analysis, 2% DCC-FBS medium was ultracentrifuged at 100,000g overnight to deplete serum exosomes before adding to cells. Small vesicles present in conditioned culture media were analysed by nanoparticle tracking, using the Nanosight LM10 system (NanoSight Ltd, Amesbury, UK), configured with a 405nm laser and a high sensitivity digital camera system (OrcaFlash2.8, Hamamatsu C11440, NanoSight Ltd, Amesbury, UK). Videos of 60 s duration were analysed by the NTA-software (version 2.2), with the minimal expected particle size set at 50 nm. Each sample was diluted in nanoparticle free water (Fresenius Kabi, Runcorn, UK), so that the concentration was between $2x10^8$ and $9x10^8$ particles/ml. The presented data, which corrects for dilution, represent the size distribution profile and concentration

values from triplicate measurements. Particle counts were normalized to DNA content of cells determined by Hoecsht 33258 staining, as described previously (4).

MicroRNA uptake experiment

Cultured hESCs were transfected with pSuperior cel-miR-39 and were then decidualized by exposure to C+M for 6 days. During the last four days of culture, the medium was not changed. Conditioned medium was collected and centrifuged at 300xg and then at 12,000xg for 30 min to remove cell debris. For embryo experiments, the medium was ultracentrifuged and the pellet was washed in PBS and resuspended in BlastAssist medium. The medium was then added to various recipient cell types in culture. 24 hours later recipient cells were washed 3 times in PBS before being harvested for RNA. Human embryos at various stages of preimplantation development, including some with breached zona pellucida, were cultured in resuspended exosome-containing medium for 16 h before lysing individually with a single cell lysis kit. QPCR was performed for cel-miR-39 on donor cells, recipient cells and embryos. Cel-miR-39 levels were normalised to endogenous U6 snRNA levels. RNA was also extracted from the last PBS wash where cel-miR-39 was not detected with qPCR. Bars represent measurements for triplicates and error bars denote SEM.

References

1 Christian, M., Pohnke, Y., Kempf, R., Gellersen, B. and Brosens, J.J. (2002) Functional association of PR and CCAAT/enhancer-binding protein beta isoforms: promoter-dependent cooperation between PR-B and liver-enriched inhibitory protein, or liver-enriched activatory protein and PR-A in human endometrial stromal cells. *Mol Endocrinol*, **16**, 141-154.

2 Chen, C., Ridzon, D.A., Broomer, A.J., Zhou, Z., Lee, D.H., Nguyen, J.T., Barbisin, M., Xu, N.L., Mahuvakar, V.R., Andersen, M.R. *et al.* (2005) Real-time quantification of microRNAs by stem-loop RT-PCR. *Nucleic Acids Res*, **33**, e179.

3 Tang, F., Hajkova, P., Barton, S.C., Lao, K. and Surani, M.A. (2006) MicroRNA expression profiling of single whole embryonic stem cells. *Nucleic Acids Res*, **34**, e9.

4 Brosens, J.J., Hayashi, N. and White, J.O. (1999) Progesterone receptor regulates decidual prolactin expression in differentiating human endometrial stromal cells. *Endocrinology*, **140**, 4809-4820.

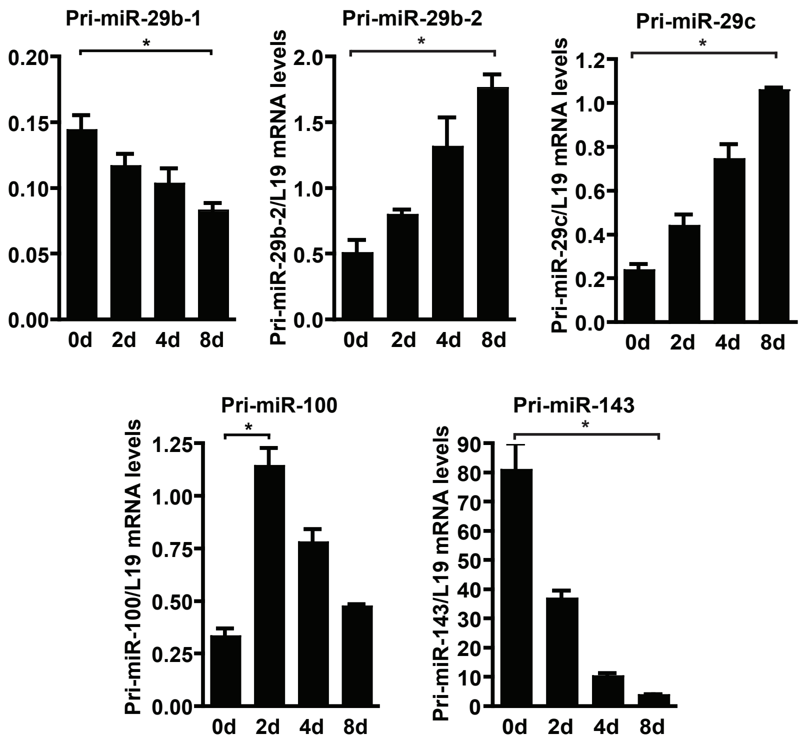

Supplementary Figure 1. Pri-miRNA levels in decidualizing hESCs
Cultured hESCs were treated with vehicle (day 0) or C+M for 2, 4 and 8 days. RNA was extracted and QPCR was used to profile the pri forms of miR-29b, miR-29c, miR-100 and miR-143. Pri-miRNA levels were normalised to L19 transcript levels. Bars represent the mean of triplicate samples and error bars denote SEM. Student's t-test was used to compare two groups of samples (usually day 0 and day 8) for significance. * $p < 0.05$.

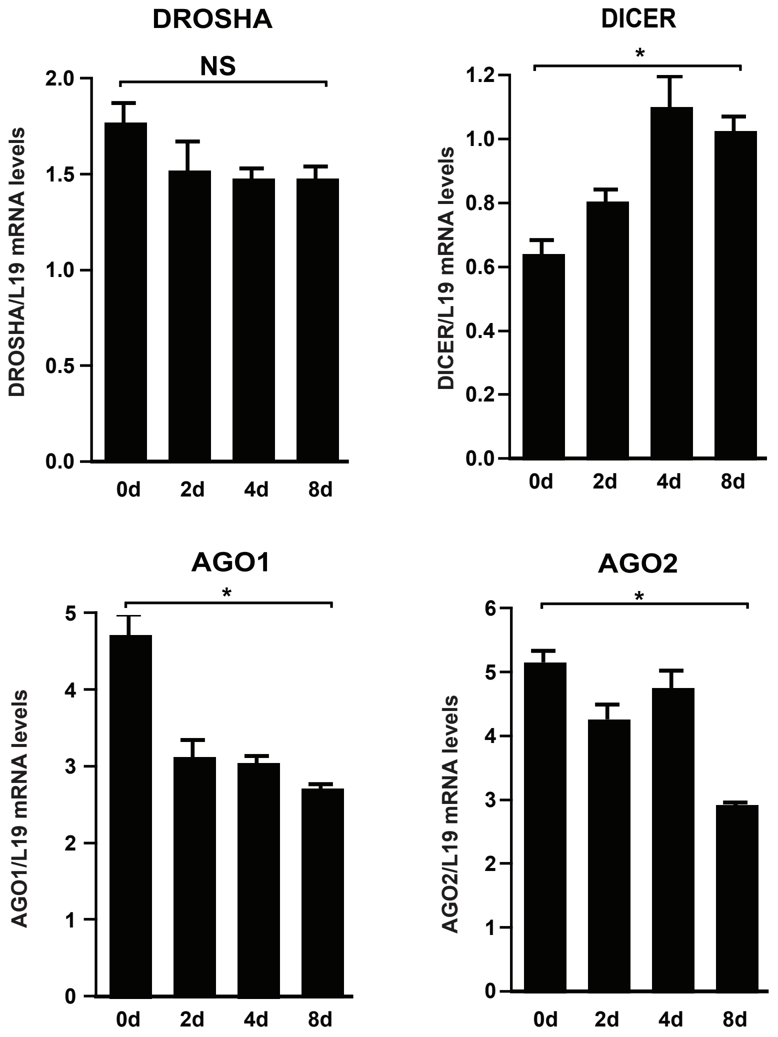

Supplementary Figure 2. mRNA levels of miRNA pathway components. Cultured hESCs were treated with vehicle (day 0) or C+M (2, 4 and 8 days). Cells were harvested for RNA. cDNA was subjected to real-time qPCR for quantification of L19, DROSHA, DICER, AGO1 and AGO2. Data were normalised to L19 expression levels. Bars show the mean of triplicate samples and error bars denote SEM. * denoted $p<0.05$ according to the student's t-test. NS indicates not significant.

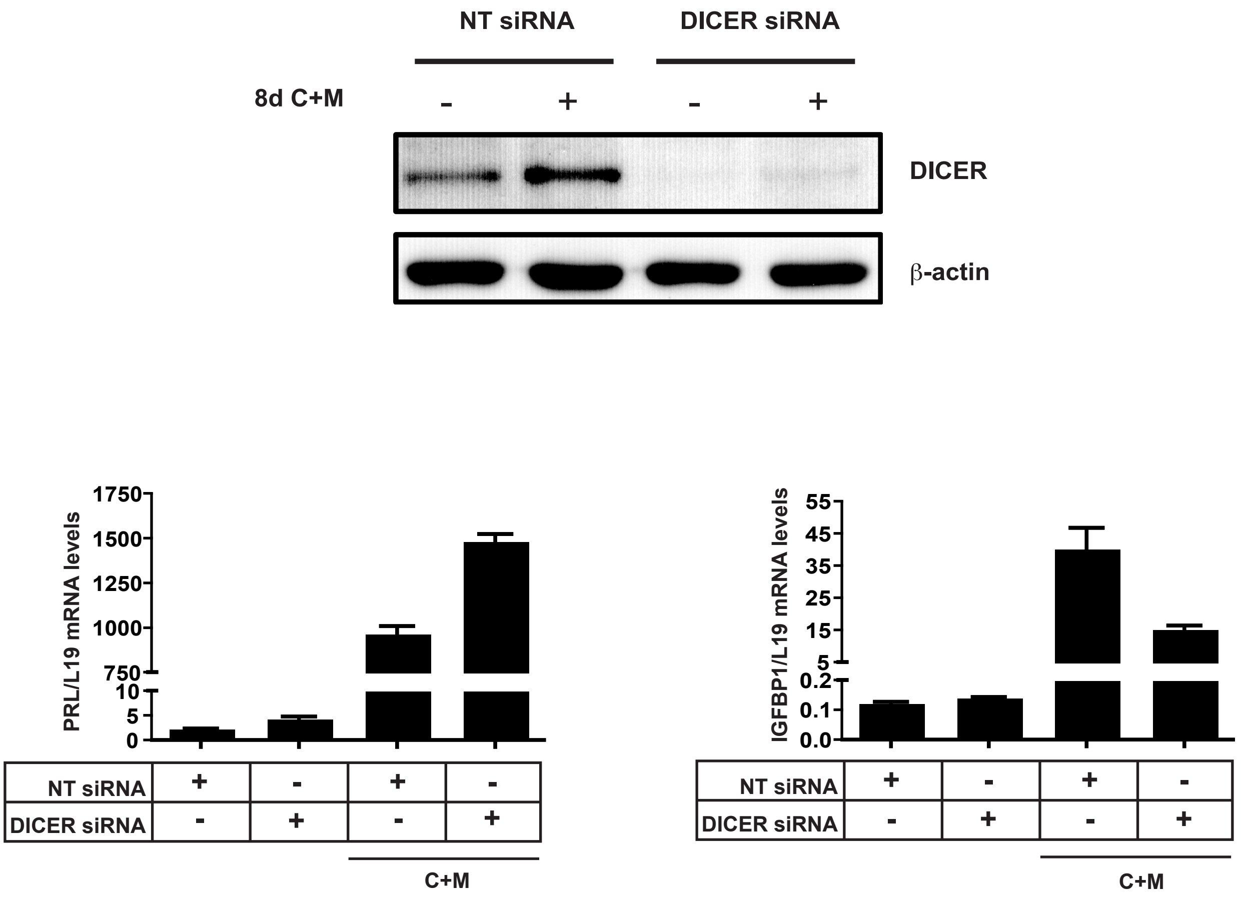

Supplementary Figure 3. The effect of Dicer knockdown on decidual marker gene expression. Cultured hESCs were treated with vehicle or C+M for 2 days and then transfected with non-targeting (NT) or DICER siRNA. Following 5 more days of treatment, cells were harvested for protein and total RNA. Protein samples were subjected to Western blotting for DICER and beta-actin as a loading control. Decidual marker gene expression was assayed with real time qPCR for PRL and IGFBP1. L19 was assayed for each sample and used to normalise the data. Error bars denote the standard error of the mean (n=3).

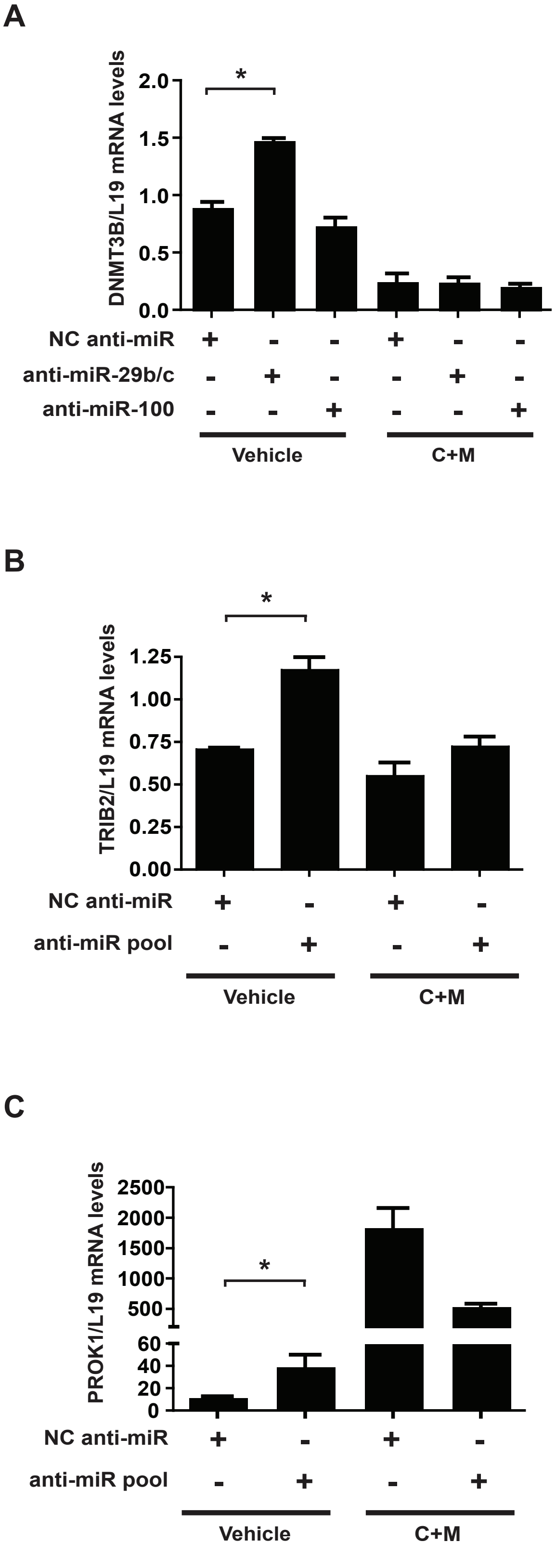

Supplementary Figure 4. Anti-miRs relieve miRNA-mediated repression of targets only in undifferentiated hESCs. (A) Cultured hESCs were transfected with negative control (NC) anti-miR, anti-miR-29b/c or anti-miR-100 and then treated with vehicle or C+M for 2 days before harvesting for RNA . DNMT3B mRNA levels were assessed by qPCR. (B and C) cultured hESCs were transfected with negative control anti-miR or a pool of anti-miRs against miR-100, miR-29b and miR-29c. Cells were then treated with vehicle or C+M for 2 days before harvesting for RNA. Expression of PROK1 and TRIB2 at the mRNA level was then determined with QPCR. For each sample L19 levels were also quantified to normalized the data. Bars represent the means of triplicate samples and error bars denote the SEM. * means that $p<0.05$ according to the Student's t-test.

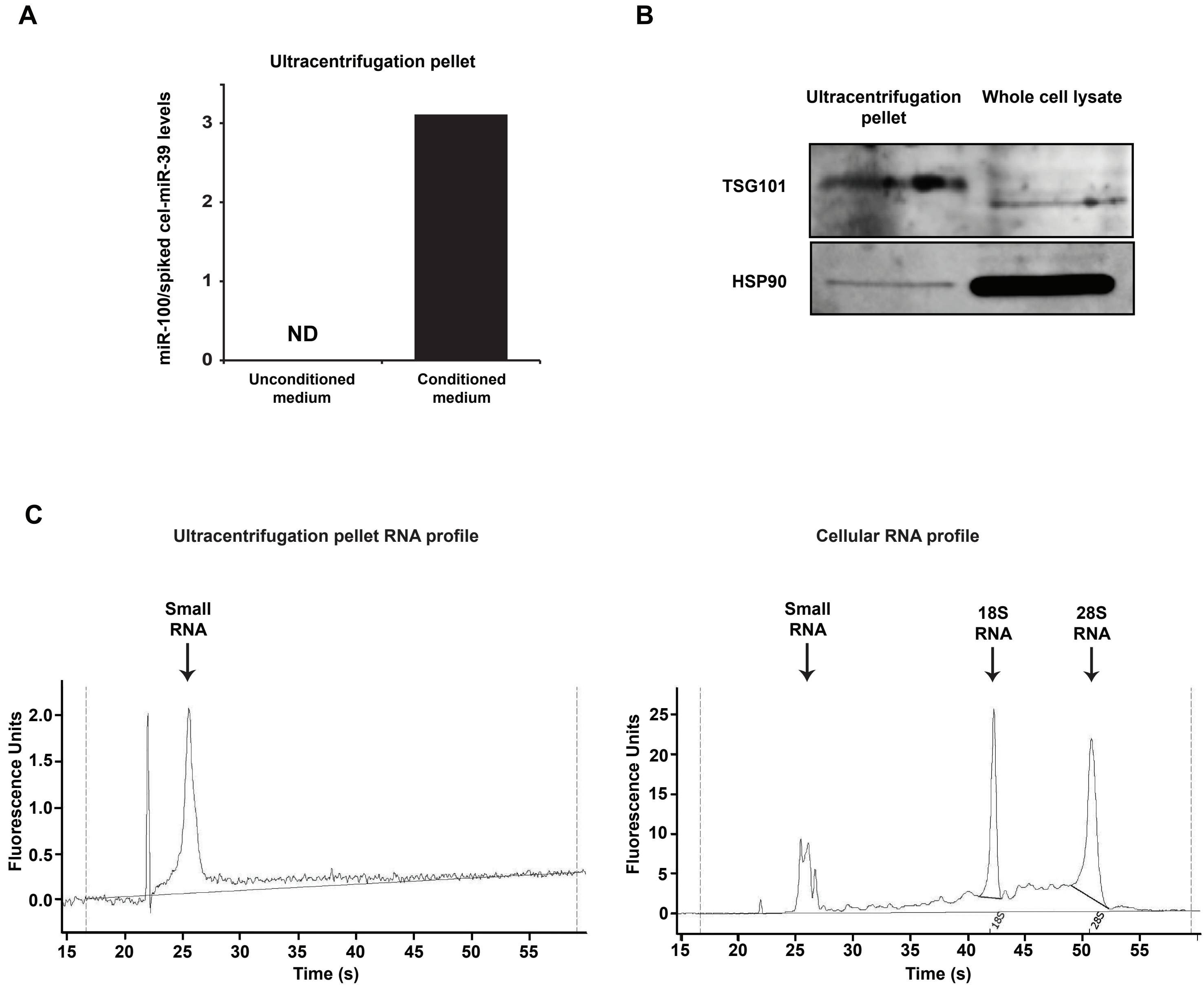

Supplementary Figure 5 Extracellular RNA in hESCs

(A) miR-100 levels were assayed in ultracentrifugation pellets of hESC conditioned medium along with spiked cel-miR-39 for normalisation. ND indicates not detected. (B) Western blots for exosome markers TSG101 and HSP90 in lysate of conditioned medium ultracentrifugation pellet or whole cell lysate from decidualized hESCs. (C) Bioanalyzer results for total RNA extracted from conditioned media ultracentrifugation pellets (left panel) or decidualized hESCs (right panel). RNA was loaded onto an RNA Pico chip and run on an Agilent 2100 Bioanalyzer according to the manufacturer's instructions.

Supplementary Table 1. Differentially expressed mature miRNAs in undifferentiated and decidualized hESCs. Triplicate small RNA samples from control undifferentiated hESCs or 8 day C+M treated hESCs were analysed using miRNA microarrays. miRNAs differentially expressed with $p < 0.05$ are listed.

miRNA	Change upon C+M Treatment	p-value
miR-143	Down	0.0072
miR-27b	Down	0.0098
let-7a	Down	0.0410
miR-377	Up	0.0390
miR-29b	Up	0.0181
miR-19b	Up	0.0219
miR-198	Up	0.0044
miR-290	Up	0.0417
miR-100	Up	0.0290
miR-30d	Up	0.0479
miR-316	Up	0.0257
miR-29c	Up	0.0072
miR-193b	Up	0.0236
miR-201	Up	0.0414
miR-22*	Up	0.0188
miR-337	Up	0.0494
